# Supplementary material for: Uniting against a common enemy: Perceived outgroup threat elicits ingroup cohesion in chimpanzees
Source: PLoS One. 2021 Feb 24;16(2):e0246869. doi: 10.1371/journal.pone.0246869 (PMC7904213; doi:10.1371/journal.pone.0246869)
Supplement: S1 File — (ZIP) [file pone.0246869.s001.zip › Playback/StabilityandColinearitySupporting.docx]

For all of our GLMMs we checked both model stability and collinearity of terms. We checked for model stability by comparing our models to those which excluded levels of the random effects one at a time. We checked for collinearity using Variance Inflation Factors (VIF, Field2005) using the function vif of the R-package car (Fox & Weisberg 2011). Output of these checks for final models reported in the main text are reported below for each model.

Stability:

Self-directed behaviours:

Playback phase:

|  | orig | min | max |
| --- | --- | --- | --- |
| (Intercept) | -4.50052 | -4.67108 | -4.40873 |
| conditionoutgroup | 1.166125 | 0.98437 | 1.419869 |
| trial_ | 0.061431 | -0.00917 | 0.135598 |
| individual_@(Intercept) | 1.133357 | 1.082783 | 1.187008 |
| individual_@conditionoutgroup | 1.200945 | 1.123592 | 1.289748 |
| individual_@trial_ | 0.776542 | 0.607865 | 0.827292 |
| individual_@conditionoutgroup:trial_ | 0.911439 | 0.67201 | 1.048086 |

Food phase:

|  | orig | min | max |
| --- | --- | --- | --- |
| (Intercept) | -4.3687 | -4.53017 | -4.26513 |
| conditionoutgroup | 0.141094 | 0.009863 | 0.366827 |
| trial_ | 0.257685 | 0.19905 | 0.35267 |
| individual_@(Intercept) | 1.262811 | 1.146611 | 1.320914 |
| individual_@conditionoutgroup | 0.806117 | 0.382431 | 0.893787 |
| individual_@trial_ | 0.69362 | 0.571545 | 0.721255 |

Social grooming:

Playback phase:

|  | orig | min | max |
| --- | --- | --- | --- |
| (Intercept) | -3.67576 | -3.84615 | -3.46682 |
| conditionoutgroup | 1.189718 | 0.90334 | 1.424945 |
| trial_ | 1.584046 | 1.230157 | 1.862863 |
| conditionoutgroup:trial_ | -1.43649 | -1.78069 | -1.08879 |
| individual_@(Intercept) | 1.313514 | 1.257741 | 1.368647 |
| individual_@conditionoutgroup | 1.286152 | 1.15144 | 1.339571 |
| individual_@trial_ | 1.206539 | 1.09679 | 1.249788 |
| individual_@conditionoutgroup:trial_ | 1.252425 | 1.163736 | 1.293375 |

Food phase:

|  | orig | min | max |
| --- | --- | --- | --- |
| (Intercept) | -4.06444 | -4.33304 | -3.87313 |
| conditionoutgroup | -0.41495 | -0.71187 | -0.13053 |
| trial_ | 0.880091 | 0.776199 | 1.027558 |
| individual_@(Intercept) | 1.32021 | 1.268743 | 1.374301 |
| individual_@conditionoutgroup | 1.559704 | 1.518803 | 1.657508 |
| individual_@trial_ | 1.057814 | 0.998398 | 1.124917 |
| individual_@conditionoutgroup:trial_ | 1.158747 | 1.030304 | 1.254549 |

Rest:

Playback phase:

|  | orig | min | max |
| --- | --- | --- | --- |
| (Intercept) | 1.132114 | 1.040647 | 1.19059 |
| conditionoutgroup | -0.44386 | -0.53715 | -0.34628 |
| trial_ | -0.60736 | -0.66664 | -0.52794 |
| conditionoutgroup:trial_ | 0.524858 | 0.429451 | 0.574275 |
| individual_@(Intercept) | 0.969455 | 0.915092 | 0.985742 |
| individual_@conditionoutgroup | 0.999086 | 0.941326 | 1.015172 |
| individual_@trial_ | 0.858158 | 0.776472 | 0.880117 |
| individual_@conditionoutgroup:trial_ | 0.76441 | 0.639725 | 0.826264 |

Food phase:

|  | orig | min | max |
| --- | --- | --- | --- |
| (Intercept) | 0.267645 | 0.23172 | 0.314244 |
| conditionoutgroup | 0.359552 | 0.306808 | 0.415513 |
| trial_ | 0.150791 | 0.126469 | 0.194543 |
| conditionoutgroup:trial_ | -0.35678 | -0.39908 | -0.28407 |
| individual_@(Intercept) | 0.720171 | 0.682759 | 0.730085 |
| individual_@conditionoutgroup | 0.780318 | 0.67908 | 0.793741 |
| individual_@trial_ | 0.617529 | 0.413729 | 0.631519 |
| individual_@conditionoutgroup:trial_ | 0.653133 | 0.523282 | 0.670372 |

Posture:

Playback phase:

|  | orig | min | max |
| --- | --- | --- | --- |
| (Intercept) | -0.10461 | -0.25221 | 0.028052 |
| conditionoutgroup | 0.354239 | 0.234282 | 0.482504 |
| trial_ | 0.094086 | 0.046377 | 0.14239 |
| conditionoutgroup:trial_ | -0.31931 | -0.37785 | -0.24006 |
| individual_@(Intercept) | 1.264138 | 1.165075 | 1.284618 |
| individual_@conditionoutgroup | 1.190679 | 1.115665 | 1.212406 |
| individual_@trial_ | 0.736679 | 0.68089 | 0.759589 |
| individual_@conditionoutgroup:trial_ | 0.696411 | 0.622109 | 0.716937 |

Food phase:

|  | orig | min | max |
| --- | --- | --- | --- |
| (Intercept) | 0.990119 | 0.87843 | 1.058221 |
| conditionoutgroup | -0.68935 | -0.79359 | -0.60213 |
| trial_ | -0.1509 | -0.23706 | -0.09839 |
| individual_@(Intercept) | 1.054882 | 0.987407 | 1.077289 |
| individual_@conditionoutgroup | 0.95522 | 0.917964 | 0.974864 |
| individual_@trial_ | 0.88094 | 0.811257 | 0.899304 |
| individual_@conditionoutgroup:trial_ | 0.904355 | 0.823731 | 0.925515 |

Aggression:

Playback phase:

|  | orig | min | max |
| --- | --- | --- | --- |
| (Intercept) | -3.14229 | -3.38487 | -3.06323 |
| conditionoutgroup | 0.566907 | 0.374608 | 0.773007 |
| trial_ | -0.64481 | -0.85348 | -0.50674 |
| individual@(Intercept) | 1.074039 | 1.040833 | 1.127805 |

Food phase:

|  | orig | min | max |
| --- | --- | --- | --- |
| (Intercept) | -1.99415 | -2.13815 | -1.92252 |
| conditionoutgroup | -1.30541 | -1.53114 | -1.16595 |
| trial_ | -0.2353 | -0.36913 | -0.10296 |
| individual@(Intercept) | 0.963291 | 0.939305 | 1.015038 |

Play:

Playback phase:

|  | orig | min | max |
| --- | --- | --- | --- |
| (Intercept) | -1.81483 | -1.92939 | -1.74831 |
| conditionoutgroup | -0.89284 | -1.04051 | -0.75985 |
| trial_ | 0.316589 | 0.255225 | 0.352063 |
| individual@(Intercept) | 0.827526 | 0.792711 | 0.870813 |

Food phase:

|  | orig | min | max |
| --- | --- | --- | --- |
| (Intercept) | -3.56964 | -3.88431 | -3.45711 |
| conditionoutgroup | 1.154664 | 0.641164 | 1.463181 |
| trial_ | -0.02338 | -0.13313 | 0.077705 |
| individual@(Intercept) | 1.328113 | 1.275844 | 1.440011 |

Collinearity:

Self-directed behaviour:

Playback phase:

condition trial_

1.000018 1.000018

Food phase:

condition trial_

1.000003 1.000003

Social grooming:

Playback phase:

condition trial_ condition:trial_

1.001096 1.992401 1.993188

Food phase:

condition trial_

1.000003 1.000003

Rest:

Playback phase:

condition trial_ condition:trial_

1.001096 1.992401 1.993188

Food phase:

condition trial_ condition:trial_

1.000086 1.998549 1.998662

Posture:

Playback phase:

condition trial_ condition:trial_

1.001464 2.120163 2.118520

Food phase:

condition trial_

1.001915 1.001915

Aggression:

Playback phase:

condition trial_

1 1

Food phase:

condition trial_

1 1

Play:

Playback phase:

condition trial_

1 1

Food phase:

condition trial_

1 1
