# Supplementary material for: Uniting against a common enemy: Perceived outgroup threat elicits ingroup cohesion in chimpanzees
Source: PLoS One. 2021 Feb 24;16(2):e0246869. doi: 10.1371/journal.pone.0246869 (PMC7904213; doi:10.1371/journal.pone.0246869)
Supplement: S1 File — (ZIP) [file pone.0246869.s001.zip › Playback/SupportingInformation-FigS1TableS1.docx]

**Graphs of significant interactions by condition and effect**


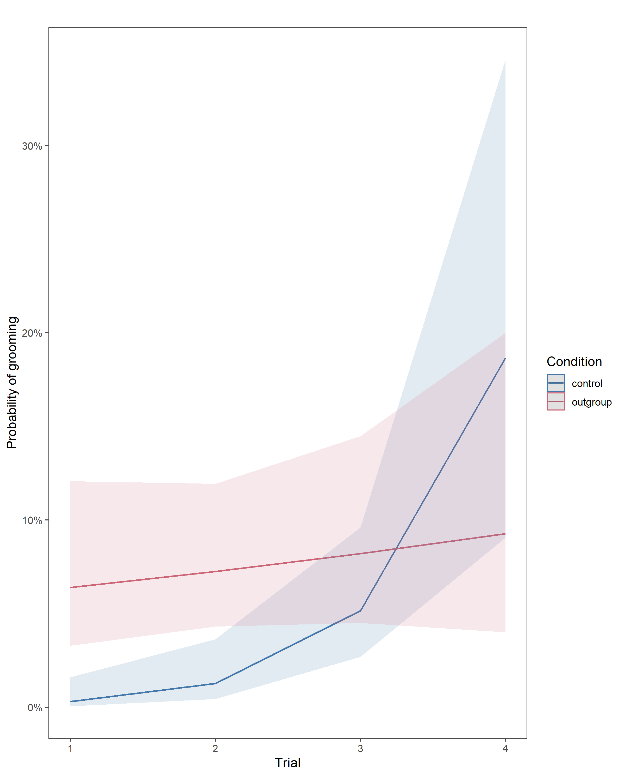

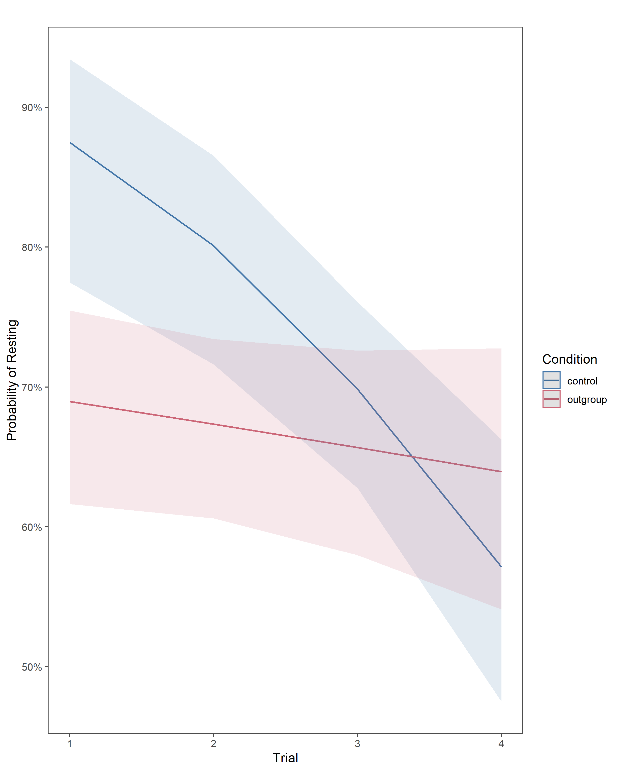

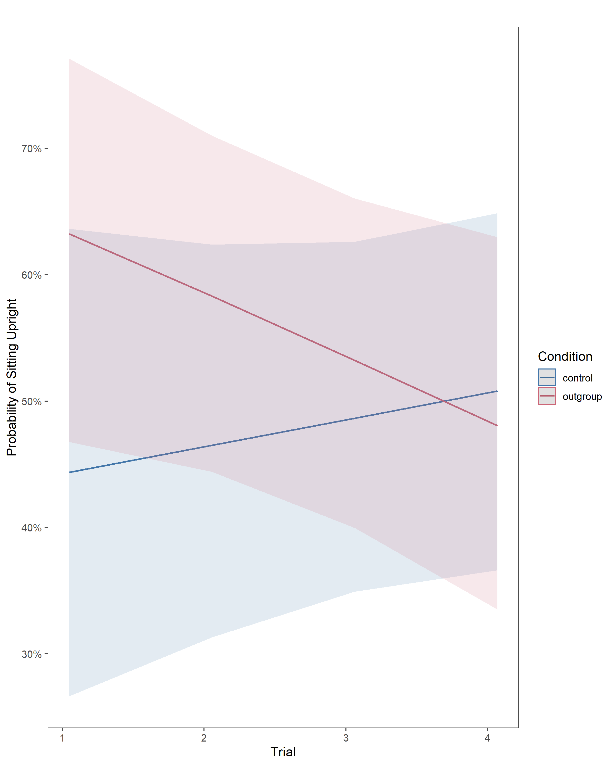

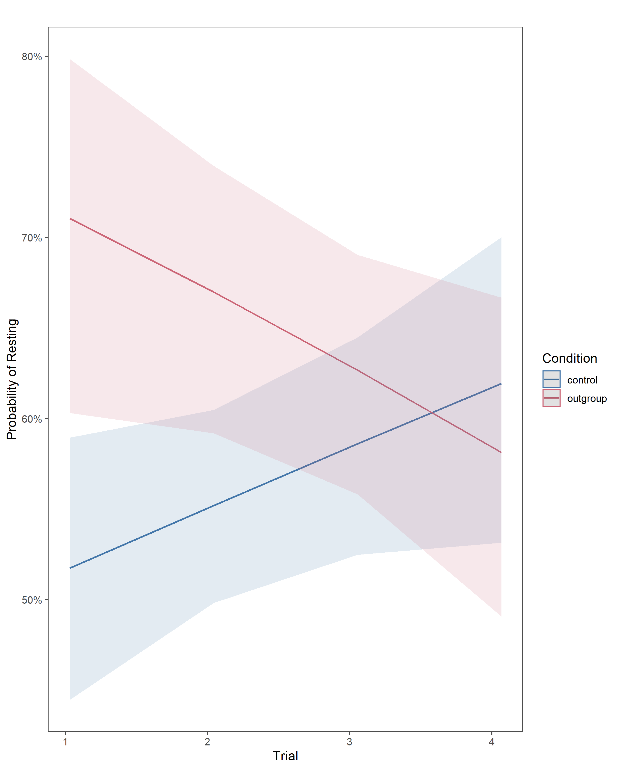


a

d

b

c

**Fig. S1.** Graphs of significant interactions between condition and trial. a) grooming in playback phase b) rest in playback phase c) sitting upright in playback phase d) rest in food phase. Daily variation is high, but both playback and controls trials were performed on the same day to control for such variation. The difference between conditions, rather than their absolute rates, therefore, are what should be attended to.

**Table of results by group**

| Group | | | 1A | 1B | 1C | 5D | 5G |
| --- | --- | --- | --- | --- | --- | --- | --- |
| Sex composition | | | 5 males | 5 males | 5 males | 1 male, 4 females | 1 male, 8 females |
| Proximity | Playback | Outgroup | 3.42 | 3.65 | 3.68 | 3.36 | 3.57 |
|  |  | Control | 3.45 | 3.55 | 3.74 | 3.46 | 3.72 |
|  | Food | Outgroup | 3.47 | 3.63 | 3.57 | 3.55 | 3.69 |
|  |  | Control | 3.61 | 3.62 | 3.74 | 3.46 | 3.78 |
| Grooming | Playback | Outgroup | 0.20 | 0.16 | 0.04 | 0.11 | 0.15 |
|  |  | Control | 0.23 | 0.18 | 0.03 | 0 | 0.11 |
|  | Food | Outgroup | 0.03 | 0.08 | 0.05 | 0 | 0.03 |
|  |  | Control | 0.06 | 0.06 | 0.04 | 0.04 | 0.08 |
| Self-directed behaviour | Playback | Outgroup | 0.08 | 0.13 | 0.02 | 0.21 | 0.02 |
|  |  | Control | 0.01 | 0.08 | 0.008 | 0.02 | 0.02 |
|  | Food | Outgroup | 0.04 | 0.07 | 0 | 0.10 | 0.04 |
|  |  | Control | 0.03 | 0.05 | 0.01 | 0.07 | 0.02 |
| Rest | Playback | Outgroup | 0.62 | 0.59 | 0.80 | 0.61 | 0.64 |
|  |  | Control | 0.62 | 0.54 | 0.75 | 0.87 | 0.73 |
|  | Food | Outgroup | 0.59 | 0.51 | 0.65 | 0.70 | 0.66 |
|  |  | Control | 0.51 | 0.56 | 0.58 | 0.63 | 0.56 |
| Sitting upright | Playback | Outgroup | 0.60 | 0.61 | 0.59 | 0.31 | 0.45 |
|  |  | Control | 0.44 | 0.59 | 0.65 | 0.39 | 0.33 |
|  | Food | Outgroup | 0.40 | 0.67 | 0.78 | 0.44 | 0.42 |
|  |  | Control | 0.69 | 0.83 | 0.70 | 0.57 | 0.56 |
| Play | Playback | Outgroup | 0.15 | 0.10 | 0.10 | 0.10 | 0 |
|  |  | Control | 0.10 | 0.05 | 0.25 | 0 | 0.03 |
|  | Food | Outgroup | 0.45 | 0.45 | 0.10 | 0 | 0 |
|  |  | Control | 0.35 | 0 | 0.10 | 0 | 0 |
| Aggression | Playback | Outgroup | 0.10 | 0.15 | 0.25 | 0 | 0.11 |
|  |  | Control | 0.25 | 0.25 | 0.45 | 0 | 0 |
|  | Food | Outgroup | 0.15 | 0.05 | 0.10 | 0 | 0 |
|  |  | Control | 0.35 | 0.20 | 0.25 | 0 | 0.06 |

**Table S1.** Table of exact values for each measure by group, phase, and condition. Proximity values represent averages of proximity categories (where 1 is in contact, 2 is arm’s reach, 3 is <3 meters, and 4 is >4 meters). Behaviour values represent absolute rates of events (as a proportion of scans or proportion of individuals who performed the behaviour in a given trial)
