# Supplementary material for: Uniting against a common enemy: Perceived outgroup threat elicits ingroup cohesion in chimpanzees
Source: PLoS One. 2021 Feb 24;16(2):e0246869. doi: 10.1371/journal.pone.0246869 (PMC7904213; doi:10.1371/journal.pone.0246869)
Supplement: S1 File — (ZIP) [file pone.0246869.s001.zip › Playback/SupportingModelResults.docx]

Maximal convergent singular models:

Singular models were overfitted and thus resulted in low significance for fixed terms compared to the final models presented in the main text. Singular models arise when the complexity of the model terms is more than can be supported by the data, and thus can result in low impact for any given variable of interest when included with many others. Model simplification is one strategy used to solve this, by removing the random effects and slopes with the least importance to the data until the model is no longer singular and can give meaningful results about the impact of each term on measures of interest. This strategy was taken in the main text, but for completeness we include singular models here.

Proximity: The models reported in the main text were the first to converge.

Behaviours: All behavioural models converged, and thus the results of the full (singular) models are shown here. The models included several predictor variables that overfit the data, and thus model simplification was necessary.

Self-directed behaviour:

Playback phase:

Model:

sd ~ condition * trial_ + (condition * trial_ + timeofday + time_ |

group_/individual_)

npar AIC LRT Pr(Chi)

<none> 1010

condition:trial_ 1 1008 0.0013347 0.9709

Model:

sd ~ condition + trial_ + (condition * trial_ + timeofday + time_ |

group_/individual_)

npar AIC LRT Pr(Chi)

<none> 1008.0

condition 1 1008.0 1.96974 0.1605

trial_ 1 1006.3 0.28034 0.5965

Food phase:

Model:

sd ~ condition * trial_ + (condition * trial_ + timeofday + time_ |

group_/individual_)

npar AIC LRT Pr(Chi)

<none> 915.35

condition:trial_ 1 913.36 0.012147 0.9122

Model:

sd ~ condition + trial_ + (condition * trial_ + timeofday + time_ |

group_/individual_)

npar AIC LRT Pr(Chi)

<none> 913.36

condition 1 911.37 0.00787 0.9293

trial_ 1 912.35 0.98120 0.3219

Social grooming:

Playback phase:

Model:

grooming ~ condition * trial_ + (condition * trial_ + timeofday +

time_ | group_/individual_)

npar AIC LRT Pr(Chi)

<none> 1625.8

condition:trial_ 1 1628.2 4.4503 0.0349 *

Food phase:

Model:

grooming ~ condition * trial_ + (condition * trial_ + timeofday +

time_ | group_/individual_)

npar AIC LRT Pr(Chi)

<none> 902.46

condition:trial_ 1 900.54 0.077006 0.7814

Model:

grooming ~ condition + trial_ + (condition * trial_ + timeofday +

time_ | group_/individual_)

npar AIC LRT Pr(Chi)

<none> 900.54

condition 1 898.51 -0.02005 1.0000

trial_ 1 900.48 1.94037 0.1636

Rest:

Playback phase:

Model:

rest ~ condition * trial_ + (condition * trial_ + timeofday +

time_ | group_/individual_)

npar AIC LRT Pr(Chi)

<none> 3228.4

condition:trial_ 1 3228.5 2.0261 0.1546

Model:

rest ~ condition + trial_ + (condition * trial_ + timeofday +

time_ | group_/individual_)

npar AIC LRT Pr(Chi)

<none> 3228.5

condition 1 3226.5 0.059294 0.8076

trial_ 1 3226.7 0.212578 0.6448

Food phase:

Model:

rest ~ condition * trial_ + (condition * trial_ + timeofday +

time_ | group_/individual_)

npar AIC LRT Pr(Chi)

<none> 4080.8

condition:trial_ 1 4078.9 0.089483 0.7648

Model:

rest ~ condition + trial_ + (condition * trial_ + timeofday +

time_ | group_/individual_)

npar AIC LRT Pr(Chi)

<none> 4078.9

condition 1 4078.7 1.7595 0.1847

trial_ 1 4078.5 1.5707 0.2101

Posture:

Playback phase:

Model:

sit ~ condition * trial_ + (condition * trial_ + timeofday +

time_ | group_/individual_)

npar AIC LRT Pr(Chi)

<none> 2683.6

condition:trial_ 1 2682.0 0.3389 0.5605

Model:

sit ~ condition + trial_ + (condition * trial_ + timeofday +

time_ | group_/individual_)

npar AIC LRT Pr(Chi)

<none> 2682.0

condition 1 2680.1 0.110061 0.7401

trial_ 1 2680.0 0.043618 0.8346

Food phase:

Model:

sit ~ condition * trial_ + (condition * trial_ + timeofday +

time_ | group_/individual_)

npar AIC LRT Pr(Chi)

<none> 2429.4

condition:trial_ 1 2427.6 0.095116 0.7578

Model:

sit ~ condition + trial_ + (condition * trial_ + timeofday +

time_ | group_/individual_)

npar AIC LRT Pr(Chi)

<none> 2427.6

condition 1 2426.1 0.56973 0.4504

trial_ 1 2425.6 0.06862 0.7934

Aggression:

Playback phase:

Model:

ag ~ condition * trial_ + (condition * trial_ + timeofday | group/individual)

npar AIC LRT Pr(Chi)

<none> 200.92

condition:trial_ 1 199.07 0.1533 0.6954

Model:

ag ~ condition + trial_ + (condition * trial_ + timeofday | group/individual)

npar AIC LRT Pr(Chi)

<none> 199.07

condition 1 197.73 0.6548 0.41839

trial_ 1 200.63 3.5542 0.05939 .

Food phase:

Model:

ag ~ condition * trial_ + (condition * trial_ + timeofday | group/individual)

npar AIC LRT Pr(Chi)

<none> 182.29

condition:trial_ 1 179.75 -0.53812 1

Model:

ag ~ condition + trial_ + (condition * trial_ + timeofday | group/individual)

npar AIC LRT Pr(Chi)

<none> 179.75

condition 1 188.51 10.7572 0.001039 **

trial_ 1 180.73 2.9794 0.084328 .

Play:

Playback phase:

Model:

play ~ condition * trial_ + (condition * trial_ + timeofday |

group/individual)

npar AIC LRT Pr(Chi)

<none> 203.30

condition:trial_ 1 203.26 1.9597 0.1615

Model:

play ~ condition + trial_ + (condition * trial_ + timeofday |

group/individual)

npar AIC LRT Pr(Chi)

<none> 203.26

condition 1 202.00 0.7401 0.3896

trial_ 1 202.45 1.1955 0.2742

Food phase:

Model:

play ~ condition * trial_ + (condition * trial_ + timeofday |

group/individual)

npar AIC LRT Pr(Chi)

<none> 189.35

condition:trial_ 1 187.61 0.25785 0.6116

Model:

play ~ condition + trial_ + (condition * trial_ + timeofday |

group/individual)

npar AIC LRT Pr(Chi)

<none> 187.61

condition 1 185.62 0.01312 0.9088

trial_ 1 186.14 0.53538 0.4644
