# Supplementary figures and images for: Uniting against a common enemy: Perceived outgroup threat elicits ingroup cohesion in chimpanzees
Source: PLoS One. 2021 Feb 24;16(2):e0246869. doi: 10.1371/journal.pone.0246869 (PMC7904213; doi:10.1371/journal.pone.0246869)

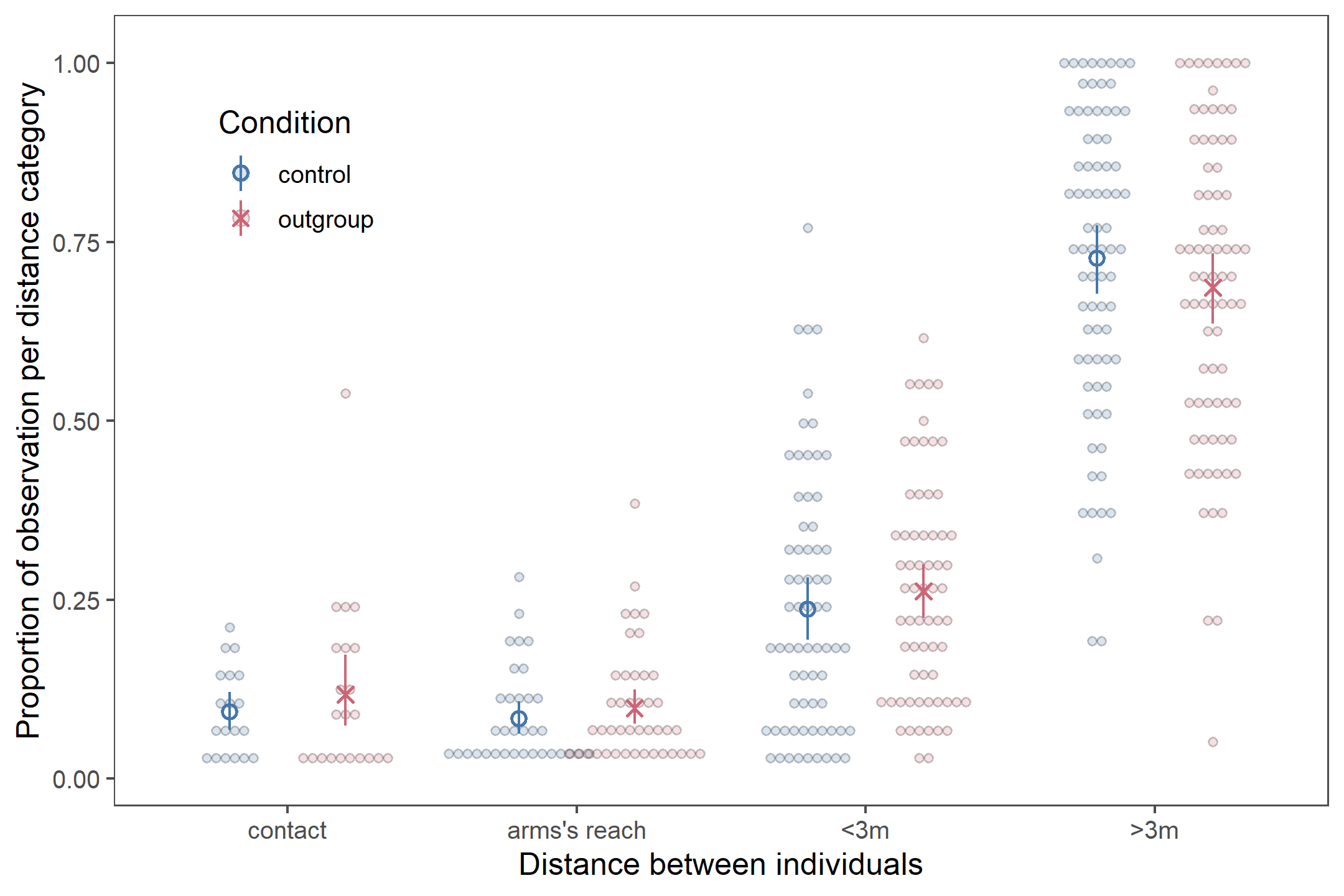

Supplement: S1 File — (ZIP) [file pone.0246869.s001.zip › Playback/figures/Fig1.tiff]

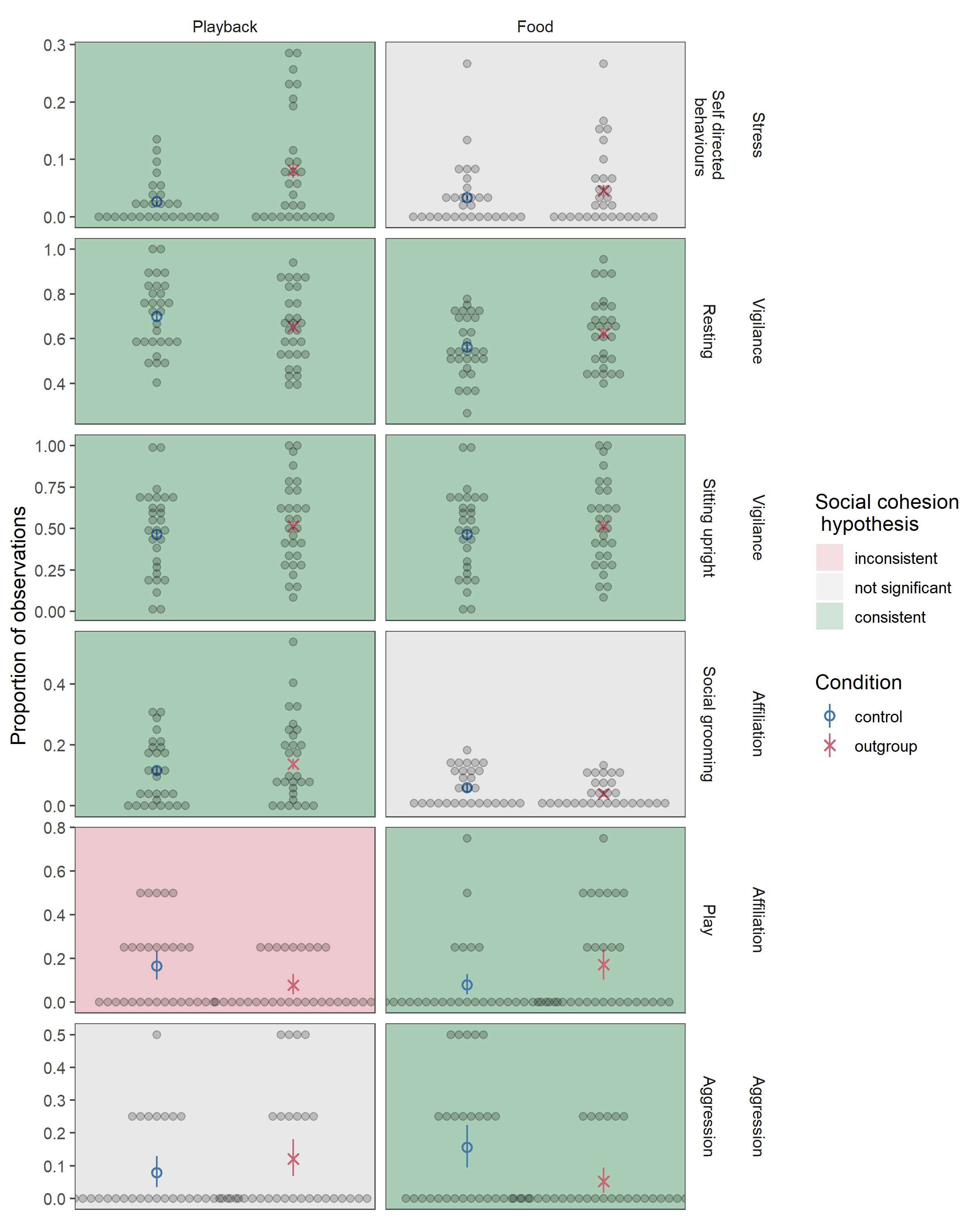

Supplement: S1 File — (ZIP) [file pone.0246869.s001.zip › Playback/figures/Fig2.tiff]

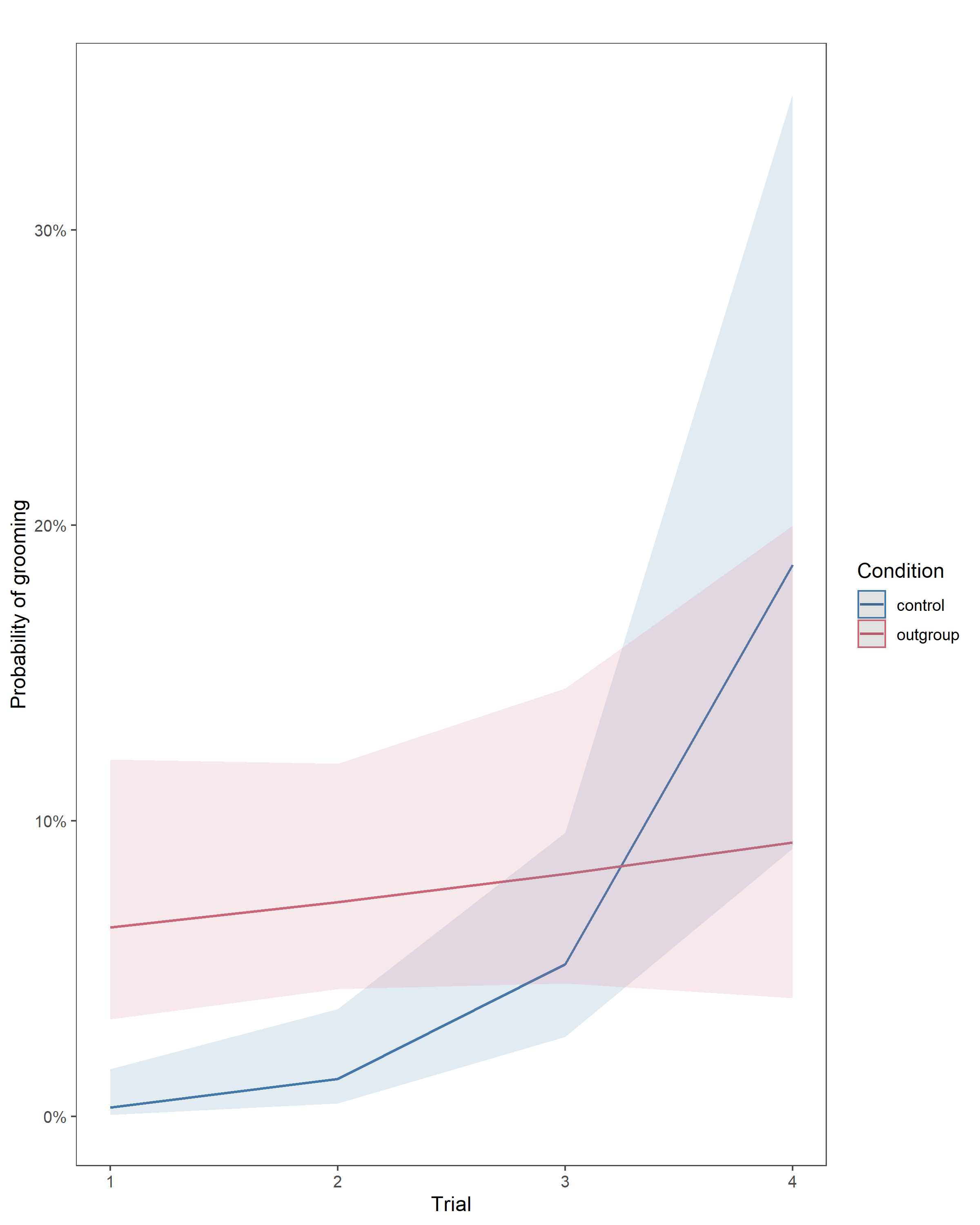

Supplement: S1 File — (ZIP) [file pone.0246869.s001.zip › Playback/figures/FigS1a.tiff]

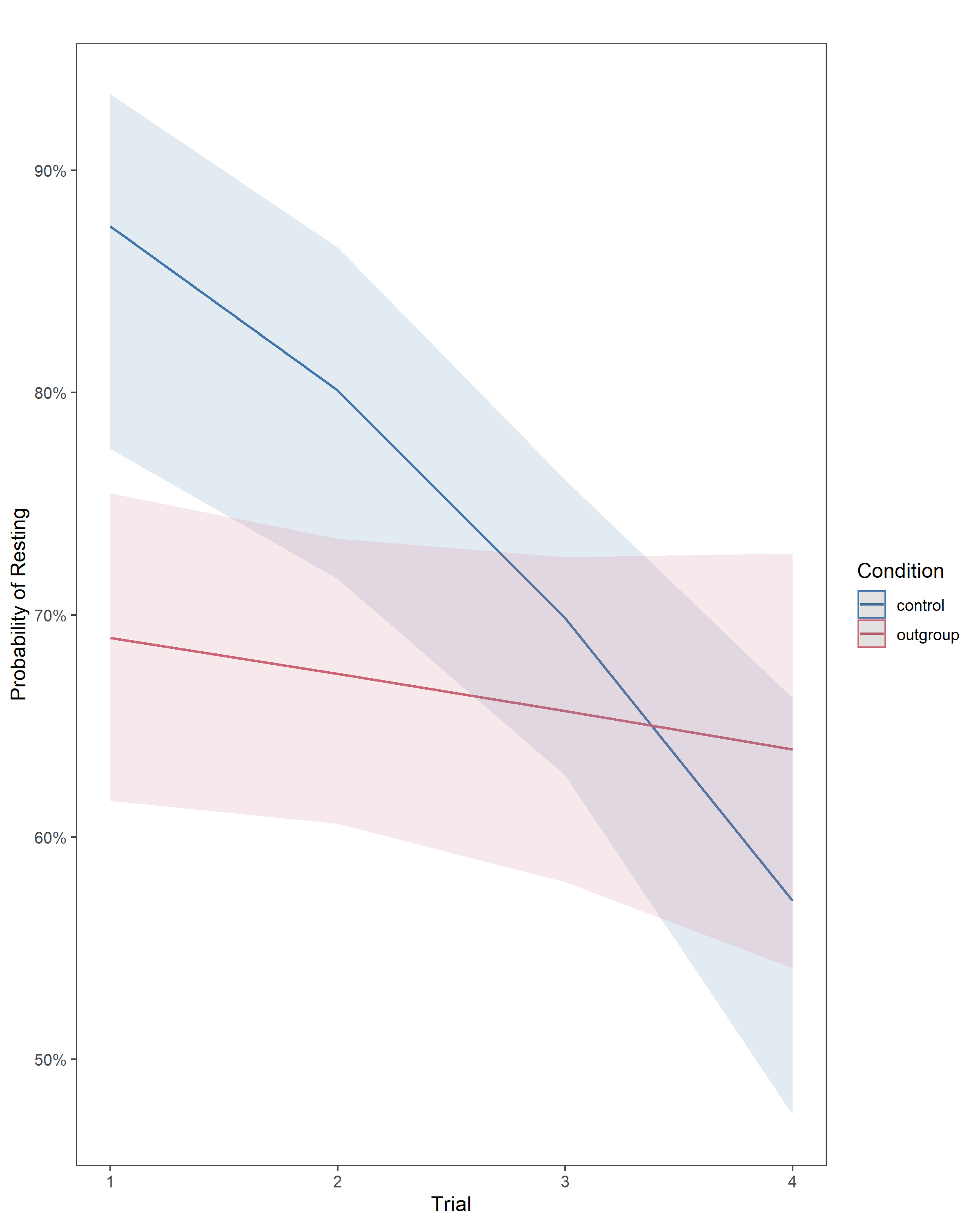

Supplement: S1 File — (ZIP) [file pone.0246869.s001.zip › Playback/figures/FigS1b.tiff]

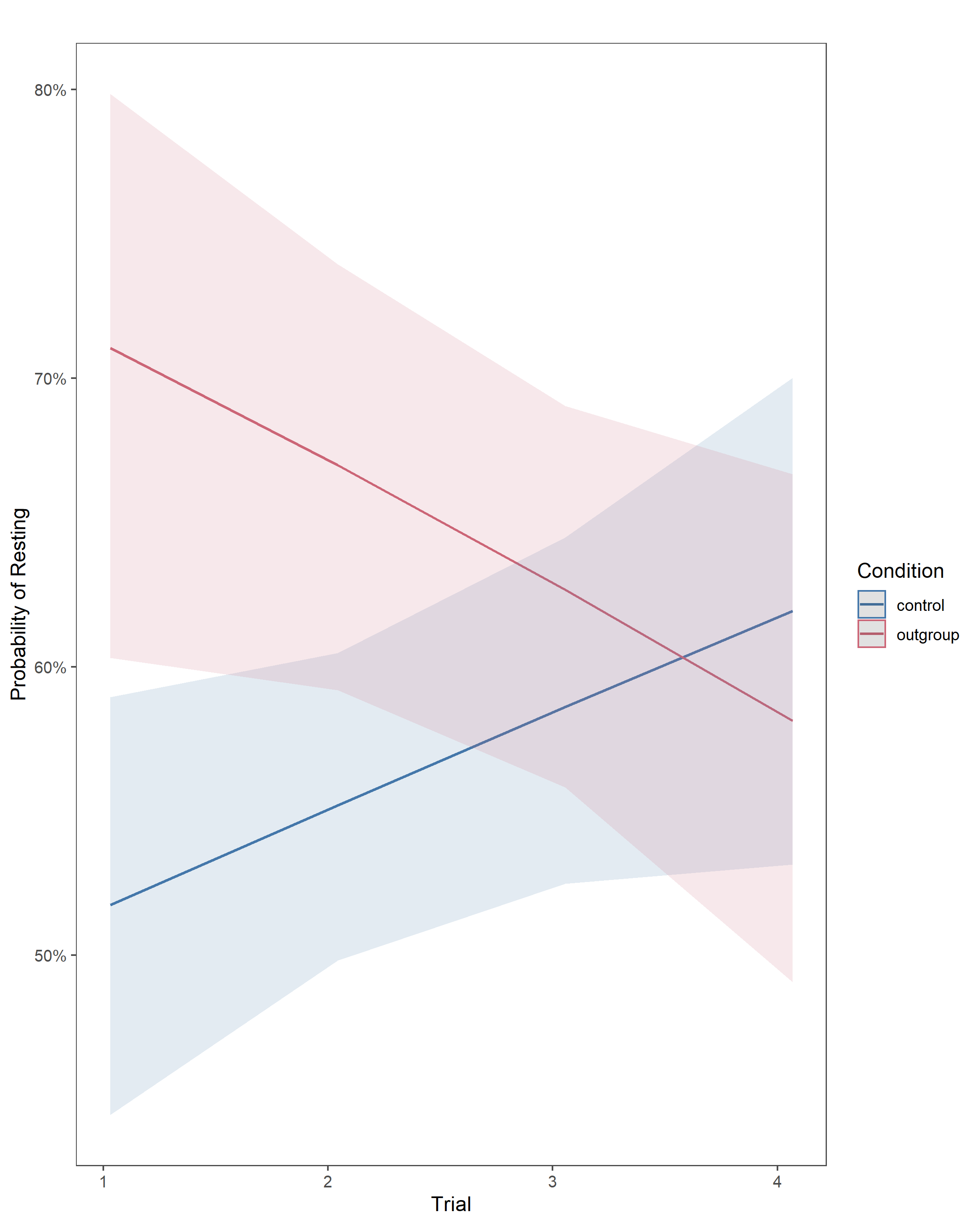

Supplement: S1 File — (ZIP) [file pone.0246869.s001.zip › Playback/figures/FigS1c.tiff]

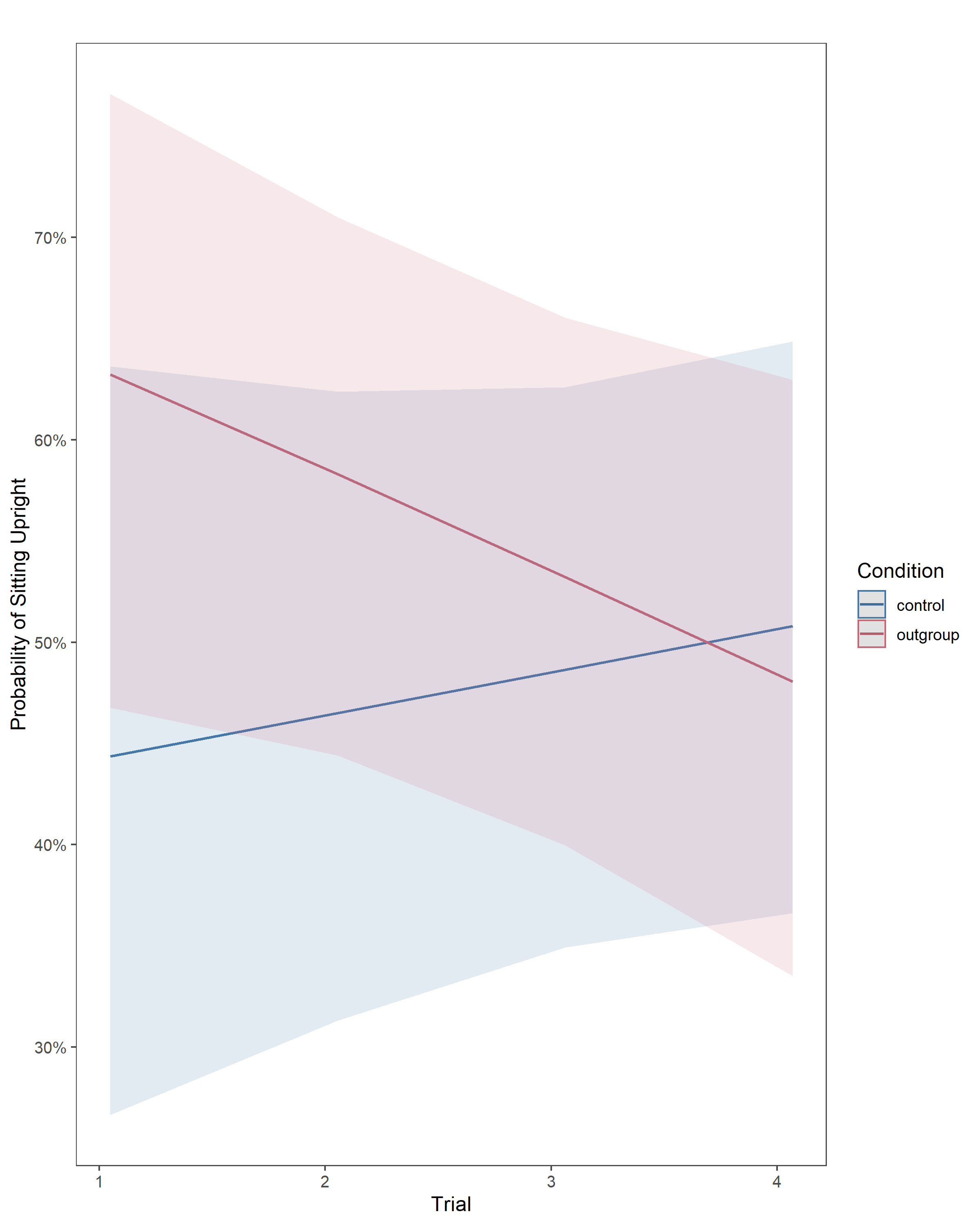

Supplement: S1 File — (ZIP) [file pone.0246869.s001.zip › Playback/figures/FigS1d.tiff]
