# Supplementary material for: Uniting against a common enemy: Perceived outgroup threat elicits ingroup cohesion in chimpanzees
Source: PLoS One. 2021 Feb 24;16(2):e0246869. doi: 10.1371/journal.pone.0246869 (PMC7904213; doi:10.1371/journal.pone.0246869)
Supplement: Attachment — Submitted filename: editorreply_rev2.docx [file pone.0246869.s003.docx]

Dear Professor Slocombe,

Thank you very much for your feedback and the chance to resubmit. We agree will all your suggestions, and have made the relevant changes to the manuscript and have added the requested figures to supplementary information. We additionally revised minor grammar and formatting points, and added a citation to a recently published study validating oxytocin administration affects social behaviour in bonobos and chimpanzees (Brooks et al. 2021). We have pasted your comments below with specific response in blue. Thank you for your consideration of our manuscript.

Sincerely,

James Brooks, Ena Onishi, Isabelle R. Clark, Manuel Bohn, and Shinya Yamamoto

Thank you for your thorough and thoughtful revision of your manuscript. I think you did an excellent job of addressing the reviewer’s comments and this is shaping up to be a great paper. Careful reading of your revision have yielded a few more areas I would like you to address. All line numbers refer to the track changed version of the manuscript.

1. Interactions. Whilst your predictions exclusively deal with main effects of condition, many of your models reveal significant interactions. Whilst you are careful to describe the pattern of interaction between trial and condition in the text you don’t provide any illustration of these effects (Figure 2 which is cited represents main effects only).

This is an important point. We have now made new visualizations for all of the effects for which the interaction was significant in order to clarify for the readers exactly how the effect changed across trials. These can be found in the supporting information zip file.

I would like you to:

i. produce figures of graphing trial and condition for each of the significant interactions and include them in the supplementary and cite them in the main text;

We have added these to the supporting information and refer to them on lines 367-369 400-401, and 425-426.

ii. Include some reference to habituation across trials and an interaction with trial being expected in the predictions section of the introduction – so making it clear that either a main effect of condition or an interaction with trial and condition, where the effect of condition decreases with trial would be supportive of the hypothesis (so that Figure 2 can legitimately say the effect is ‘consistent’ with the hypothesis even when it represents an interaction with trial).

We have added mention of this aspect of our hypotheses on lines 163-166.

iii. Be explicit in the figure 2 legend that the measures which produced significant interactions are graphed here as main effects of condition and refer the reader to the new figures in the supplementary if they wish to see the interactions graphed.

We have added this information (lines 367-369).

iv. I am unsure of how to interpret an oddsratio for an interaction – could help the reader to understand what it means?

Thank you for pointing this out, odds ratios are less straightforward to interpret with interactions and we have described this on lines 289-293.

2. Ethical concerns. Some readers may find the increase in stress the experimental procedure induced ethically questionable. I think it would be good to highlight in the discussion that investigation of a natural event which induces stress (intergroup encounters) is impossible without elevating stress levels, but as there was no difference in stress behaviours in the feeding context, the induced stress effects appeared to be shortlived (30 minutes), and therefore do not represent a substantial welfare issue.

Thank you for mentioning this point, we have added these details into the methods in the subsection “Ethical note” in order to give readers a clearer understanding of our ethical thinking (lines 171-178).

3. In response to R1, you now state in the discussion there was considerable group variation. Please provide some data to demonstrate this in the supplementary (maybe figures showing the effect of condition by group?) and add a short section in the results to introduce this. Group doesn’t make it as a factor in most of your final models, so currently you don’t provide any evidence to support your claim of group variation, and it would be good for the interested reader to be able to look at the variation.

Although we do not have sufficient data to include group type as a factor in our models, we recognize the importance of this and have added to the supporting information a table which include values for each group and measure to demonstrate the variation, and added mention of this to the manuscript in lines 360-363 and 479.

L471-3 I’m not quite clear what the point you are trying to make here – what part of the results could these factors account for (unless its clear what they might explain, saying they are unlikely to explain it doesn’t make much sense!).

We have taken out this sentence. We had no specific predictions regarding how it could account for any part of our results, but mentioned it simply to clarify potential sources of variation. As you note it made the paragraph more confusing and we removed this sentence. Thank you for your suggestion.

L512 – insert ‘they’ between ‘chimpanzees’ and ‘do’.

Done

Thank you again for your careful consideration and advice on our manuscript.
